# A novel scoring system tailored for rabbit models of endotoxin-induced uveitis: feasible, sensitive, and reproducible

**DOI:** 10.1186/s12886-026-04611-4

**Published:** 2026-01-24

**Authors:** Lin Kang, Yu Jin, Feiyue Xiao, Wubi Li, Chan Zhao

**Affiliations:** 1 Biomedical Engineering Facility of National Infrastructures for Translational Medicine, Institute of Clinical Medicine, Peking Union Medical College Hospital, Chinese Academy of Medical Sciences and Peking Union Medical College, Beijing, 100730 China; 2 Department of Ophthalmology, Peking Union Medical College Hospital, Chinese Academy of Medical Sciences and Peking Union Medical College, Beijing, 100730 China; 3 Key Laboratory of Ocular Fundus Diseases, Chinese Academy of Medical Sciences and Peking Union Medical College, Beijing, 100730 China

**Keywords:** Endotoxin-induced uveitis, Rabbit, Clinical scoring criteria, Anterior segment photography, Triamcinolone acetonide

## Abstract

**Background:**

Uveitis is an inflammatory ocular disease that can lead to vision impairment. Endotoxin-induced uveitis (EIU) models are commonly used in animal studies to investigate acute uveitis for drug screening and understanding disease mechanisms. However, most clinical scoring systems for EIU were developed for rat models. Applying these rat-derived criteria to rabbit EIU models may introduce inaccuracies due to interspecies differences between rabbit and rat eyes, potentially affecting the precision of inflammation assessment. Therefore, the aim of this study is to establish a novel clinical scoring system for rabbit EIU models.

**Methods:**

In this study, a novel quantitative scoring system based on anterior chamber photography, using iris hyperemia, anterior chamber exudates, and hypopyon as key indicators, was established based on the clinical manifestations of EIU in rabbits.

**Results:**

Utilizing triamcinolone acetonide (TA) as a model drug, the treatment groups showed significantly lower scores in rabbit EIU compared to control groups at different stages. This novel scoring system effectively reflected the therapeutic efficacy of varying TA concentrations. In contrast, the classical rat-derived scoring system was less sensitive in distinguishing disease activity at different levels of immunosuppression. The interobserver consistency was strong with a kappa value of 0.796.

**Conclusion:**

This novel EIU scoring system, tailored specifically for rabbits, demonstrates excellent feasibility, sensitivity, and reproducibility.

## Background

Uveitis is a group of ocular inflammatory disorders affecting the uveal tract and/or adjacent structures, including the retina, optic nerve, and vitreous [1–3]. It is one of the leading causes of blindness, particularly among young adults. Each year, up to 5–10% of uveitis patients worldwide experience severe visual impairment or legal blindness [4–6]. Prompt management of acute uveitis or acute flare-ups of chronic uveitis is essential to prevent complications and irreversible vision loss [7, 8]. Due to the limited availability of ocular tissues for research, animal models of uveitis are essential for studying the disease’s mechanisms and evaluating potential treatments.

A major advancement in the development of uveitis animal models was made by Rosenbaum et al., who demonstrated that systemic immunization with bacterial lipopolysaccharide (LPS) induces acute anterior uveitis in rats, establishing the endotoxin-induced uveitis (EIU) model [9]. Subsequently, their group discovered that intravitreal injection of LPS could elicit a substantial acute uveitic response in mice [10]. Since then, rodent models of EIU have predominantly favored the intravitreal route. The EIU rodent model mimics many immunopathogenic mechanisms observed in human acute uveitis and has been instrumental in developing novel therapeutic approaches [11–13]. Rats and rabbits are commonly used animals to construct EIU models [14, 15]. The severity of uveitis in rats can be quantified by clinical scores based on slit-lamp examination or anterior segment photography, following a standard grading scale [16].

In previous studies, the scoring system originally developed for rat EIU models has often been directly applied to the evaluation of rabbit EIU models [17, 18]. However, it is important to consider the anatomical differences between rats and rabbits, including variations in eyeball size, ocular vascular distribution, and iris structure [19]. These differences mean that the scoring criteria used for rat EIU models may not be fully applicable to rabbit models. Furthermore, the existing scoring system is relatively simplistic and may lack the sensitivity needed to accurately distinguish between varying severities of the disease, potentially limiting its effectiveness in assessing treatment outcomes [16]. Given these differences and limitations, there is a clear need to establish a more refined and widely accepted scoring system specifically for EIU in rabbits.

Based on the clinical features of uveitis in rabbit eyes, we developed a new scoring system that incorporates three key indicators: iris hyperemia, anterior chamber exudates, and hypopyon. In addition, statistical analysis of the efficacy of triamcinolone acetonide (TA) in treating EIU in New Zealand white rabbits demonstrated that the newly developed scoring system has excellent feasibility, sensitivity, and reproducibility. This system addresses the gap in evaluating rabbit uveitis and provides an important methodological standard and reference for future studies using rabbit EIU models.

## Materials and methods

### Materials

A total of 39 male New Zealand white rabbits, 2 to 3-month-old and weighing between 2.0 and 3.0 kg, were purchased from Beijing Vital River Laboratory Animal Technology Co., Ltd. (Beijing, China). The rabbits were housed and managed by the Experimental Animal Center of Peking Union Medical College Hospital. All animal experiments were approved by the ethical committee of Peking Union Medical College Hospital (No.XHDW-2022-100) and conducted in strict adherence to the Association for Research in Vision and Ophthalmology (ARVO) Statement for the Use of Animals in Ophthalmic and Vision Research [20]. After the experiments, all animals were euthanized by intravenous injection of pentobarbital sodium (100 mg/kg) via the marginal ear vein, resulting in rapid loss of consciousness and cardiac arrest.

Experimental treatments included: ketamine and xylazine, provided by the Experimental Animal Center of Peking Union Medical College Hospital; Tropicamide Phenylephrine Eye Drops and Oxybuprocaine Hydrochloride Eye Drops purchased from Santen Pharmaceutical Co., Ltd. (Osaka, Japan); LPS from *Salmonella enterica* serotype typhimurium purchased from Sigma-Aldrich (St. Louis, USA); TA injectable suspension Triesence^®^ (40 mg/mL) purchased from Harrow Pharmaceuticals (Alcon, Ft. Worth, TX); and sterile phosphate-buffered saline (PBS) purchased from ThermoFisher Scientific.

### Induction of rabbit EIU models

The rabbit EIU models were induced by intravitreal injection of LPS as described previously [21, 22]. In brief, rabbits were anesthetized with an intramuscular injection of ketamine and xylazine. Once anesthetized, Tropicamide Phenylephrine Eye Drops were administered to dilate the eyes, and after several minutes, the Oxybuprocaine Hydrochloride Eye Drops were applied for ocular surface anesthesia. A 30-gauge needle attached to a 1 mL syringe was carefully inserted at an oblique angle into the eye, approximately 1 mm posterior to the limbus, ensuring that no intraocular structures, such as the lens, were compromised. 50 µL of LPS (2 µg/mL) was then injected into the vitreous cavity. Upon needle withdrawal, a sterile cotton swab was gently applied to the entry point to prevent leakage.

### Drug intervention of EIU

Identical doses of LPS were administered to both the left (OS) and right (OD) eyes of each rabbit. In Experiment 1 (*n* = 15), four hours (h) post-LPS injection, 50 µL of Triesence^®^ (containing 40 mg/mL of TA) was intravitreally injected into the right eye, while an equivalent volume of PBS was injected into the left eye. In Experiment 2 (*n* = 12), the therapeutic effects of TA at high (40 mg/mL) and low (30 mg/mL) concentrations were compared using the same procedures. The experimental design was summarized in Table 1.

**Table 1:** The experimental design of experiment 1 and experiment 2

Experiment	Treatment		Number of animals	Observe timepoints
	OD	OS		
Experiment 1	TA (40 mg/mL), 50 µL	PBS, 50 µL	*n* = 15	24, 48, 72, 96 h
Experiment 2	TA (30 mg/mL), 50 µL	PBS, 50 µL	*n* = 12	24, 48, 72 h
	TA (40 mg/mL), 50 µL	PBS, 50 µL	*n* = 12	24, 48, 72 h

### Evaluation of EIU

The degree of inflammation in EIU was evaluated using anterior segment photographs taken at 24, 48, 72, and 96 h after induction with an LS-5 digital slit lamp (Chongqing Sunkingdom Medical Instrument Co., Ltd, China). Clinical scoring of EIU was conducted based on the criteria outlined in Table 2. For each eye at each time point, the severity of uveitis was quantified by adding the criteria scores, resulting in a total score ranging from 0 to 7, with any score above 0 indicating the presence of uveitis. Two ophthalmologists independently assessed the photographs in a blinded manner according to the established scoring criteria.

**Table 2:** Scoring criteria of EIU in the rabbit

Clinical signs		Score	Description
Iris hyperemia	Absent	0	The iris appears white, with no visible dilated capillaries, and the intricate texture of the iris remains clearly discernible.
	Mild	1	Localized dilation of capillaries is present on the surface of the iris, with partial loss of fine iris texture.
	Moderate	2	Diffuse dilation of capillaries is observed on the iris surface, resulting in the complete loss of fine iris texture and visibility of the coarser texture.
	Severe	3	The iris exhibits diffuse hyperemia and edema, making the iris texture difficult to distinguish.
Anterior chamber exudates	Absent	0	No exudates is observed from the pupillary margin, lens, or surface of the iris.
	Mild	1	A small amount of jelly-like exudates cover < 1/4 of the pupillary margin, or fibrous exudates appear.
	Moderate	2	Increased jelly-like exudates, which obscure < 1/2 of the pupillary margin.
	Severe	3	Jelly-like exudates obscure ≥ 1/2 of the pupillary margin.^a^
Hypopyon	Absent	0	
	Present	1	White, clumpy sediment in the anterior chamber.^b^
Maximum score		7	

^a^ Iris hyperemia scores highest in eyes with severe anterior chamber exudates

^b^ The top scores are assigned to the first two parameters in eyes with hypopyon

### Statistical analysis

Each anterior segment photograph was independently scored by two observers, and the average score was used as the final score for each treatment and time point in each experiment. Statistical analyses were conducted using SPSS version 29.0. Two-way ANOVA with Tukey post hoc test was used to analyze the statistical differences among multiple groups. *P* < 0.05 was considered statistically significant. Cohen’s kappa (κ) test was utilized to assess interobserver agreement, and a κ value > 0.6 indicating good agreement.

## Results

### Development of the novel scoring criteria for rabbit EIU model

**Fig. 1:**
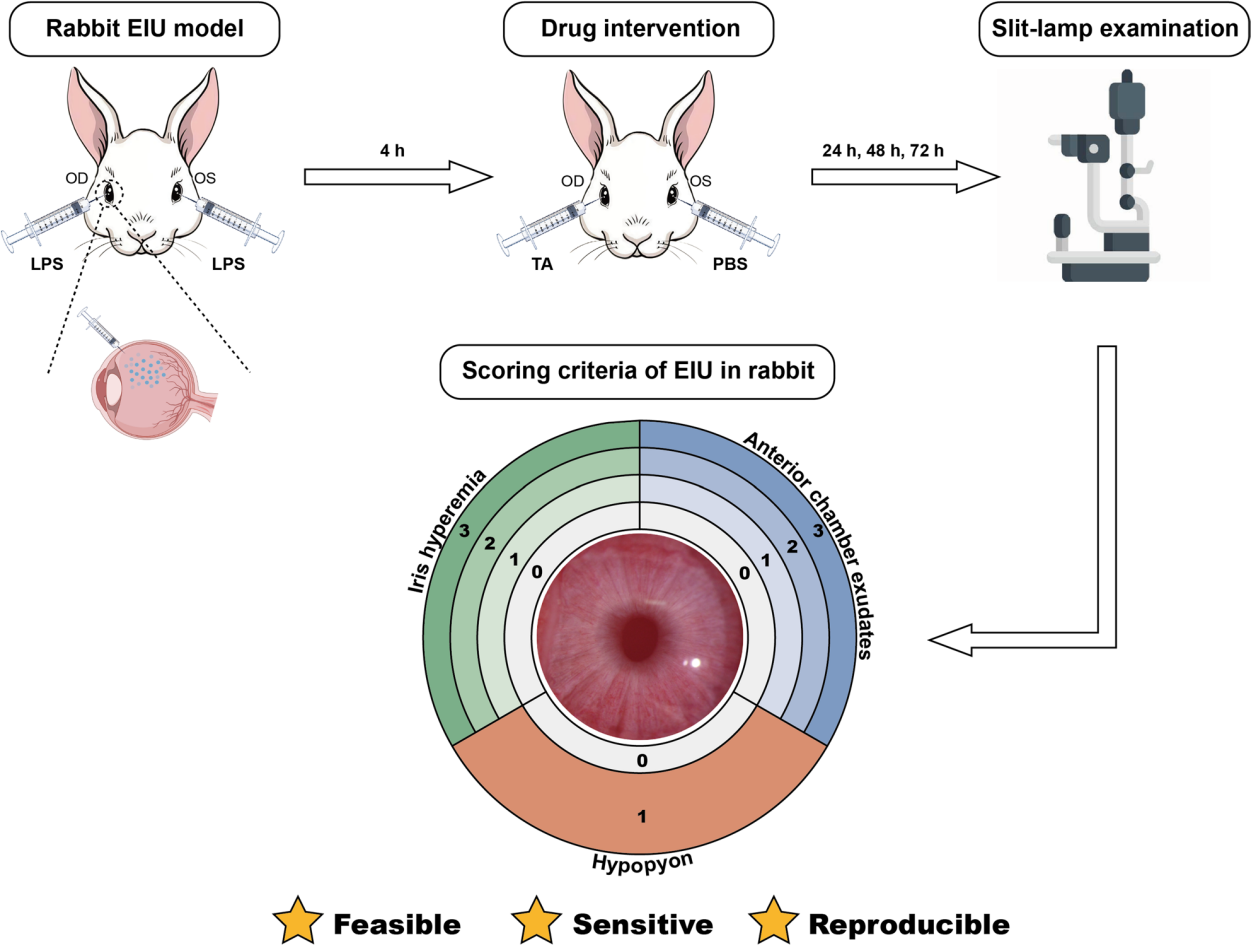
Schematic diagram of the experimental procedure and the novel scoring system for rabbit EIU models. The rabbit EIU model was induced in New Zealand white rabbits via intravitreal injection of LPS. Four hours post-injection, the contralateral eyes received an equal volume of either PBS or triamcinolone acetonide (TA) as an intervention. Anterior segment images were captured using a slit lamp at various time points thereafter. The severity of uveitis was quantified by summing the scores for iris hyperemia, anterior chamber exudates, and hypopyon. Both iris hyperemia and anterior chamber exudates were graded on a scale of 0 to 3 (0: none, 1: mild, 2: moderate, 3: severe). Hypopyon was scored in a binary manner (0: absent, 1: present). This novel scoring system, tailored specifically for rabbit EIU models, demonstrates excellent feasibility, sensitivity, and reproducibility

**Fig. 2:**
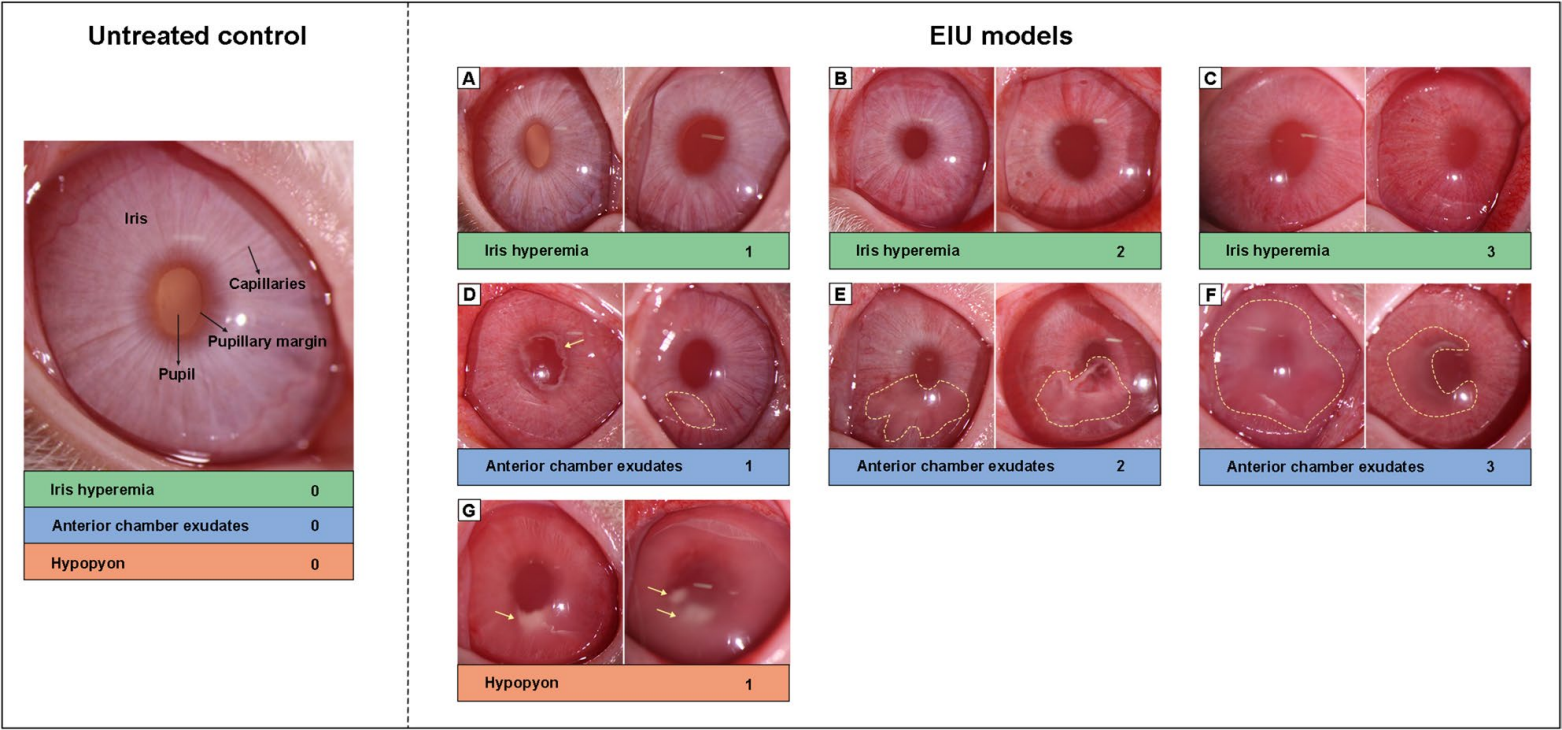
Representative photographs and corresponding scores of the rabbit anterior segment showing varying degrees of iris hyperemia, anterior chamber exudates, and hypopyon in the EIU models. For an untreated control, the iris exhibits a white appearance with no visible dilated capillaries and exudates. For EIU models, mild iris hyperemia with localized dilation of capillaries (**A**); moderate iris hyperemia with diffuse dilation of capillaries, loss of fine iris texture and visibility of the coarser texture (**B**); severe iris hyperemia exhibited diffuse hyperemia and edema of iris, with difficult distinguished iris texture (**C**); mild anterior chamber exudates, characterized by a small amount of jelly-like exudations covering < 1/4 of the pupillary margin, or the presence of fibrous exudates (**D**); moderate exudates with jelly-like exudates covering < 1/2 of the pupillary margin (**E**); severe exudates with jelly-like exudates covering ≥ 1/2 of the pupillary margin (**F**); hypopyon with white clumpy sediments in the anterior chamber (**G**); images were randomly selected from all experimental eyes, yellow arrows and dotted line indicated typical lesion locations

Based on longitudinal observations of disease course with and without intravitreal TA treatments, we identified iris hyperemia, anterior chamber exudates, and hypopyon as the key clinical features of rabbit EIU. Unlike in rat EIU, where miosis is a prominent clinical sign, it was neither consistently present in rabbits nor was it associated with disease severity. Consequently, we developed a novel scoring system to assess rabbit EIU, by evaluating iris hyperemia, anterior chamber exudates, and hypopyon. As illustrated in Fig. 1, iris hyperemia and anterior chamber exudates were scored as follows: no hyperemia/exudates, mild, moderate, and severe, assigned scores of 0, 1, 2, and 3, respectively. The presence of hypopyon received a score of 1, and its absence received a score of 0. Notably, iris hyperemia received the highest score in eyes with severe anterior chamber exudates, while in eyes with hypopyon, both iris hyperemia and anterior chamber exudates received the highest score. Detailed descriptions of the scoring criteria for each feature are provided in Table 2, and representative photographs of the rabbit anterior segment for each scoring scenario are displayed in Fig. 2.

### Evaluating the therapeutic effect on rabbit EIU using the scoring system

**Fig. 3:**
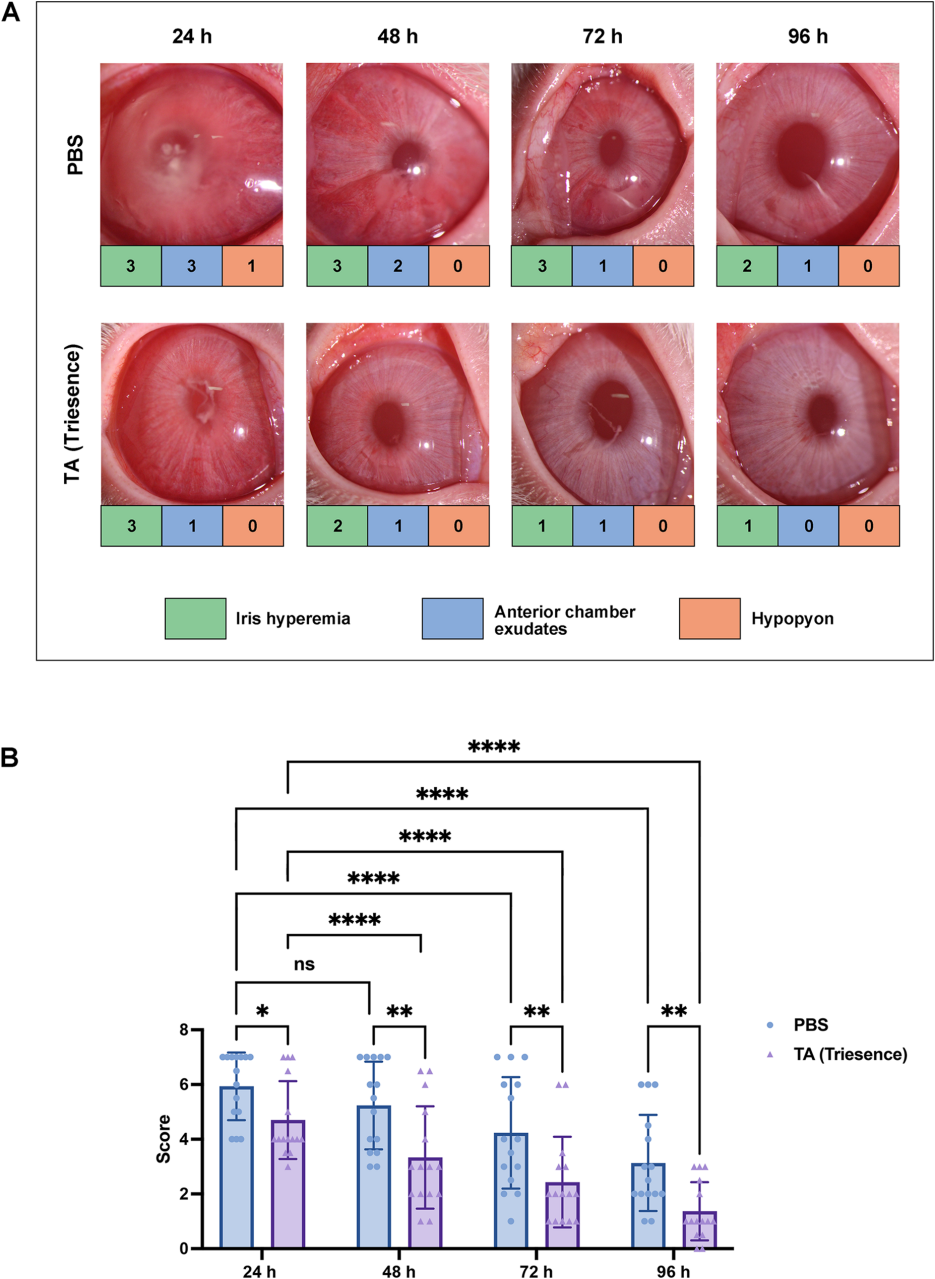
Evaluation of the efficacy of TA in rabbit EIU models in Experiment 1. (**A**) Representative anterior segment photographs of the rabbits at 24, 48, 72, and 96 h after 40 mg/mL TA (OD) or PBS (OS) intervention; (**B**) Statistical results of the mean group scores in Experiment 1 according to the new scoring system at different time points. Statistical analysis was conducted using two-way ANOVA for multiple groups, *n* = 15, ^*^P < 0.05, ^**^P < 0.01, ^****^P < 0.0001

To evaluate the effectiveness of our new EIU scoring system, we used TA suspension (Triesence^®^), a known therapeutic agent for uveitis [23, 24], as the treatment group, comparing it to a PBS control group. Triesence is a preservative-free formulation of TA [25]. As a corticosteroid, TA works by inhibiting the production and release of inflammatory mediators and suppressing the activation and migration of inflammatory cells. This mechanism effectively reduces the dilation of capillaries and limits fibrin exudation, thereby alleviating the clinical findings associated with uveitis [26, 27]. Representative photographs of the anterior segment for both the treatment and control groups at 24, 48, 72, and 96 h are shown in Fig. 3A. Statistical analysis, presented in Fig. 3B, demonstrated that the scores in the TA-treated group were significantly lower than those in the control group at various time points (^*^P < 0.05, ^**^P < 0.01). Additionally, the scores in the TA-treated group progressively decreased over time, with scores at 48, 72, and 96 h significantly lower than those at 24 h (^****^P < 0.0001). The PBS group also exhibited a declining trend in scores over time, reflecting the self-limiting course of the EIU model. These results indicate that the new EIU scoring system correlates well with the severity of inflammation in rabbits.

### Assessment of differences in efficacy through the scoring system

**Fig. 4:**
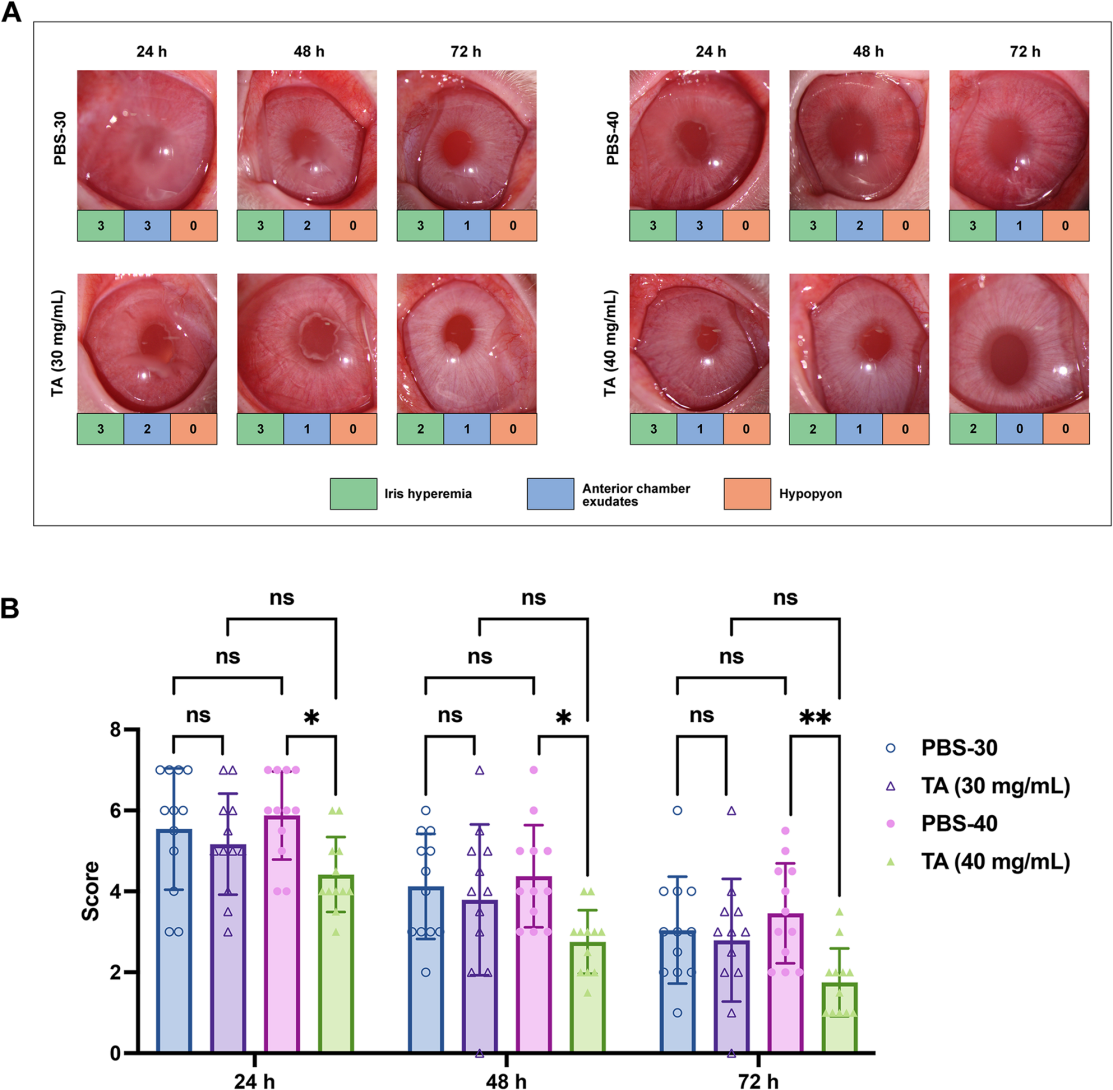
Therapeutic effects of high and low concentrations of TA on uveitis in rabbit EIU in Experiment 2. (**A**) Representative anterior segment photographs of the rabbits treated with high (40 mg/mL) and low (30 mg/mL) concentrations of TA (OD) against PBS (OS); (**B**) Statistical results of the mean group scores in Experiment 2 according to the new scoring system with high (40 mg/mL) and low (30 mg/mL) concentrations of TA at different time points. Statistical analysis was conducted using two-way ANOVA for multiple groups, *n* = 12, ^*^P < 0.05, ^**^P < 0.01

To further evaluate the accuracy and discriminatory ability of this new scoring system, we validated it using high and low concentrations of TA. Representative photographs and scores of rabbit anterior segments at both concentrations, compared to the PBS control, are shown in Fig. 4A. As shown in Fig. 4B, statistical analysis revealed that although the mean scores of the TA-30 group (30 mg/mL) were lower than those of the PBS group at all observation time points, the differences were not statistically significant. In contrast, the mean scores of the TA-40 group (40 mg/mL) were significantly lower than those of the PBS group at 24, 48, and 72 h (^*^P < 0.05, ^**^P < 0.01). These findings demonstrate that the new scoring system effectively reflects differences in the extent of inflammation inhibition by different drug concentrations, suggesting that it possesses a strong discriminatory ability for EIU models.

### Comparison of the classical and the new scoring systems

**Fig. 5:**
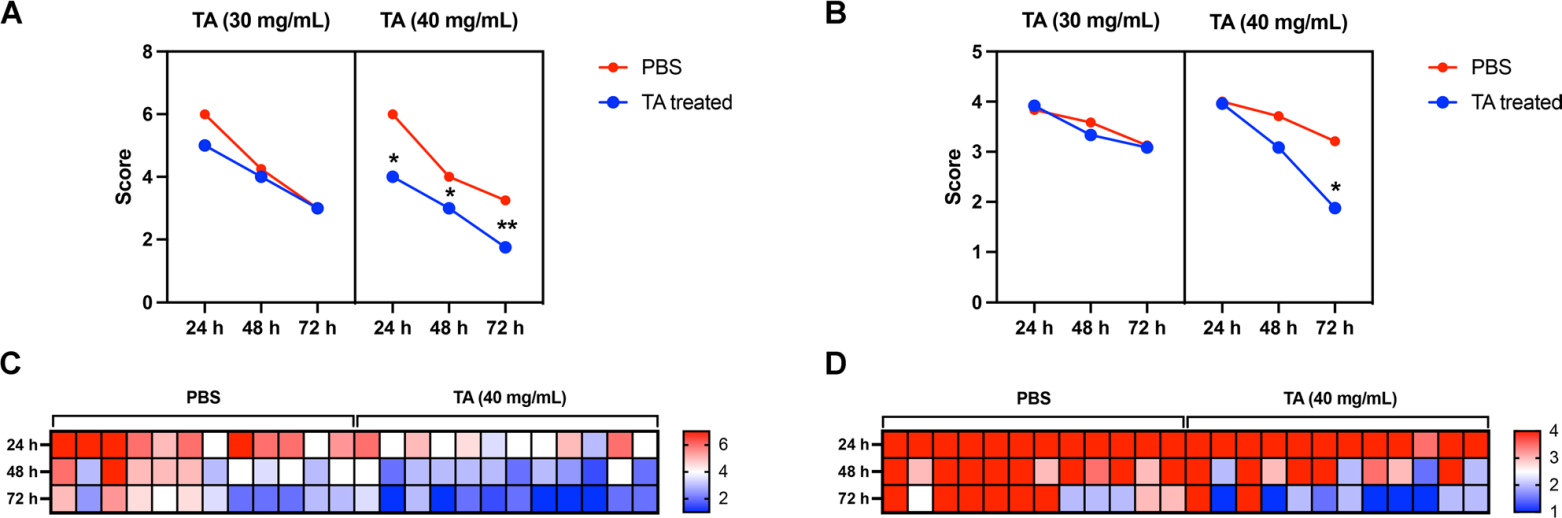
Comparison of the classical and the new scoring systems within Experiment 2. (**A**) Clinical score curves of rabbit eyes according to the new scoring system; (**B**) Clinical score curves of rabbit eyes using the classical scoring criteria; (**C**) Heatmap of scores assigned by the new scoring system in rabbit EIU models treated with 40 mg/mL TA; (**D**) Heatmap of the TA-treated rabbit EIU models assessed by the classical scoring system. Statistical analysis was conducted using two-way ANOVA for multiple groups, *n* = 12, ^*^P < 0.05, ^**^P < 0.01

We conducted a comparative analysis between the new scoring system and the classical scoring system described by Ruiz-Moreno et al., which categorized the severity of inflammation in the EIU model on a scale of 0 to 4 [16, 28]. The results demonstrated that the new scoring system exhibited superior sensitivity compared to the classical system. Specifically, at TA concentration of 40 mg/mL, the scores determined by the new criteria were significantly lower than those of the PBS group at 24, 48, and 72 h (Fig. 5A) (^*^P < 0.05, ^**^P < 0.01). In contrast, the scores assigned by the classical scoring system showed no statistically significant differences between the treated group and the control group at 24 and 48 h (Fig. 5B). Additionally, heatmap analysis revealed that the classical scoring system tended to cluster at higher scores, making it difficult to distinguish severity of early uveitis in the rabbit EIU models. On the other hand, the new scoring system provided relatively more granular and differentiated evaluation criteria for uveitis, by incorporating three distinct criteria (Fig. 5C and D).

### The consistency of the scoring system

We evaluated the consistency of the scores assigned by two observers according to the new scoring system. The results showed a high level of agreement between the observers across multiple experimental trials. This concordance was evident not only in the overall scores (κ = 0.796) but also in the individual assessments of iris hyperemia (κ = 0.799), anterior chamber exudates (κ = 0.785) and hypopyon (κ = 0.896). Detailed statistical results are presented in Table 3. These results demonstrated that the new scoring system for evaluating EIU models in rabbits had good repeatability.

**Table 3:** The agreement between the two observers

Category	Interobserver agreement (κ/*p*)		
	Sample 1^a^	Sample 2^b^	Total^c^
Iris hyperemia	0.857 / <0.001	0.741 / <0.001	0.799 / <0.001
Anterior chamber exudates	0.795 / <0.001	0.773 / <0.001	0.785 / <0.001
Hypopyon	0.853 / <0.001	0.956 / <0.001	0.896 / <0.001
Overall	0.815 / <0.001	0.777 / <0.001	0.796 / <0.001

^a^ Scoring values for all samples from Fig. 3

^b^ Scoring values for all samples from Fig. 4

^c^ Scoring values combined Fig. 3 with Fig. 4

## Discussion

The most commonly used animal model of acute anterior uveitis is the EIU model induced by intravitreal injection of LPS [29, 30]. Slit-lamp examination or photography of the anterior segment is a non-invasive and sustainable method valued for its simplicity and ease of use. In this study, we identified iris hyperemia, anterior chamber exudates, and hypopyon as hallmark signs of rabbit EIU, with their severity varying depending on the stage of disease. Based on these observations, we proposed this novel scoring system specifically for rabbit EIU models. Our investigations demonstrated that this scoring system effectively illustrated the disease course of EIU, which typically begins 4 h after LPS injection, peaks at 18 to 24 h, and persists for at least 72 h [13, 31, 32]. Our system also clearly distinguished between the efficacy of high and low concentrations of TA (40 mg/mL vs. 30 mg/mL) (Fig. 4). These findings suggest that the novel scoring system for rabbit EIU models is both feasible and sensitive. Furthermore, statistical analysis showed strong agreement between two independent observers, both in total scores and in individual assessments of iris hyperemia, exudates, and hypopyon (Table 3). These results demonstrate that our scoring system has strong reliability and reproducibility.

In our newly developed scoring system for rabbit EIU models, both anterior chamber exudates and hypopyon have been identified as key hallmark signs. While both occur during the acute exudative phase of inflammation, they exhibit distinct characteristics. In terms of composition, anterior chamber exudates mainly consist of serous protein and fibrinous exudation, whereas hypopyon is composed primarily of tissue debris, inflammatory by-products, and recruited leukocytes [33]. Clinically, exudates are usually transparent to translucent in appearance, as observed in aqueous “flare” or “plastic”, whereas hypopyon presents as a white, clumpy sediment, as illustrated in Fig. 2. This comparison provides valuable insights for researchers utilizing this scoring system.

In previous studies, the classical scoring system has been a widely used standard for evaluating EIU models [18, 34]. This system grades EIU severity on a scale from 0 to 4: Grade 0 indicates no apparent inflammatory response; Grade 1 indicates discrete inflammation of the iris and conjunctival vessels; Grade 2 indicates moderate dilatation of the iris and conjunctival vessels with a moderate anterior chamber flare; Grade 3 indicates intense iris congestion with an intense flare in the anterior chamber; Grade 4 indicates the same clinical signs as Grade 3 plus fibrinous exudates or pupillary constriction [13, 16]. However, our observations in the rabbit EIU models revealed that most eyes exhibited various degrees of anterior chamber exudates in the early stages after LPS injection. As a result, these eyes were often assigned the highest score under the classical criteria, making it difficult to distinguish the severity of early-stage inflammation (Fig. 5B and D). To address this issue, we categorized anterior chamber exudates into three levels: mild, moderate, and severe. This stratification enhanced the ability to differentiate early inflammatory responses, facilitating the assessment of initial drug efficacy in rabbit EIU models (Fig. 5A and C).

Other evaluation criteria for EIU models have also been reported in the literature. For instance, Rick Hoekzema et al. proposed a set of scoring criteria to assess the degree of inflammation in rat EIU models, with pupillary miosis as a key parameter [35]. Miosis is the reflexive contraction of the sphincter muscle of the iris, resulting in a reduction in pupil size. This phenomenon has also been reported in other studies using rat EIU models [36, 37]. While this scoring system has been applied to rabbit EIU models [17], its applicability may be limited due to differences in iris physiology between the two species. The rabbit’s weaker iris sphincter results in less miosis during inflammation [38], making pupil assessment less relevant in these models. Consequently, our scoring criteria for rabbit EIU models do not include pupil assessment.

A limitation of this study is its exclusive focus on the rabbit model. Additional experiments are necessary to determine whether this scoring system can be applied to other animal models. Besides, due to the concentration limitations of the commercially available TA formulation (Triesence^®^), this study only employed two drug intervention concentrations (30 mg/mL and 40 mg/mL). Therefore, scores from rabbit EIU models treated with higher TA concentrations, such as 50 mg/mL, could not be obtained. Additionally, although the experiments included multiple internal replicate groups (e.g., PBS control, TA 40 mg/mL treatment), complete replications across Experiment 1 and Experiment 2 were not performed. Therefore, it would be valuable to independently replicate these results and to validate our novel rabbit EIU scoring system in other laboratories.

## Conclusions

In conclusion, we developed a novel scoring system specifically designed to evaluate rabbit EIU models. This system includes parameters such as iris hyperemia, anterior chamber exudates, and hypopyon to assess the extent of ocular inflammation. Each parameter is further categorized into multiple sublevels based on the severity of inflammation, resulting in a quantitative scoring system with a maximum score of 7. The newly established system demonstrated excellent feasibility, sensitivity, and reproducibility in evaluating TA-treated uveitis in rabbits. This scoring system provides a new approach for assessing rabbit EIU models and establishes an important methodological standard for drug development based on rabbit uveitis models.

## Data Availability

The datasets used and/or analyzed during the current study are available from the corresponding author upon reasonable request.
